# Trajectories of cognitive symptoms and associated factors in cancer survivors after return to work: an 18-month longitudinal cohort study

**DOI:** 10.1007/s11764-022-01190-3

**Published:** 2022-03-21

**Authors:** Johanna K. Ehrenstein, Sander K. R. van Zon, Saskia F. A. Duijts, Roy E. Stewart, Josué Almansa, Benjamin C. Amick, Sanne B. Schagen, Ute Bültmann

**Affiliations:** 1 Community and Occupational Medicine, Department of Health Sciences, University Medical Center Groningen, University of Groningen, Hanzeplein 1, PO Box 30.001, 9700 RB Groningen, The Netherlands; 2 Division of Psychosocial Research and Epidemiology, The Netherlands Cancer Institute, Plesmanlaan 121, 1066 CX Amsterdam, The Netherlands; 3 Department of Public and Occupational Health, Amsterdam Public Health Research Institute, Amsterdam UMC, Vrije Universiteit Amsterdam, Van der Boechorststraat 7, 1081 BT Amsterdam, The Netherlands; 4 Department of Research and Development, Netherlands Comprehensive Cancer Organisation (IKNL), Godebaldkwartier 419, 3511 DT Utrecht, The Netherlands; 5 Department of Epidemiology, Fay W. Boozman College of Public Health, University of Arkansas for Medical Sciences, Little Rock, AR USA; 6 Department of Psychology, University of Amsterdam, Nieuwe Achtergracht 129-B, 1018 WT Amsterdam, The Netherlands

**Keywords:** Cancer, Cancer-related cognitive impairment, Employment, Rehabilitation, Quality of life, Psycho-oncology

## Abstract

**Purpose:**

Cognitive symptoms affect cancer survivors’ functioning at work. To date, cognitive symptoms trajectories in working cancer survivors and the factors associated with these trajectories have not been examined.

**Methods:**

Data from a heterogeneous group of working cancer survivors (*n* = 379) of the longitudinal “Work-Life-after-Cancer” study, linked with Netherlands Cancer Registry data, were used. The Cognitive Symptom Checklist-Work was administered at baseline (within the first 3 months after return to work), 6-, 12-, and 18-month follow-up to measure self-perceived memory and executive function symptoms. Data were analyzed using group-based trajectory modeling.

**Results:**

Four trajectories of memory and executive function symptoms were identified. All memory symptoms trajectories were stable and labeled as “stable-high” (15.3% of the sample), “stable-moderately high” (39.6%), “stable-moderately low” (32.0%), and “stable-low” (13.0%). Executive function symptoms trajectories changed over time and were labeled as “increasing-high” (10.1%), “stable-moderately high” (32.0%), “decreasing-moderately low” (35.5%), and “stable-low” (22.4%). Higher symptoms trajectories were associated with older age, longer time from diagnosis to return to work, more quantitative work demands, and higher levels of depressive symptoms at baseline.

**Conclusions:**

In cancer survivors who returned to work, four cognitive symptoms trajectory subgroups were identified, representing different but relatively stable severity levels of cognitive symptoms.

**Implications for Cancer Survivors:**

To identify cancer survivors with higher symptoms trajectories, health care providers should assess cognitive symptoms at baseline after return to work. In case of cognitive symptoms, it is important to also screen for psychological factors to provide appropriate guidance.

**Supplementary Information:**

The online version contains supplementary material available at 10.1007/s11764-022-01190-3.

## Introduction

Cancer-related cognitive impairment (CRCI) is common among cancer patients and may persist over time [1, 2]. In a recent review, it was found that approximately 40% of cancer patients have CRCI before any treatment, up to 75% may have CRCI during treatment, and up to 60% exhibit CRCI after completion of therapies [1]. CRCI can be measured via neuropsychological testing and/or self-report assessments [3]. Earlier research has shown that self-reported cognitive symptoms affect cancer survivors’ functioning at work, work ability, and work performance [4–6]. The cognitive domains most likely to be negatively affected are memory and executive functioning, but other aspects of memory and processing speed may be affected as well [7]. After return to work (RTW), memory problems may influence a working cancer survivor’s capacity to acquire the skills and knowledge necessary to carry out work-related functions [8], and executive function problems may diminish the planning and implementing of strategies.

Previous studies showed that in working cancer survivors, self-reported complaints of memory and executive function remain stable and persist over time [9, 10]. These observations are often based on aggregated information [11]. Memory and executive function symptoms may, however, be heterogeneous over time within working cancer survivors, which means that cancer survivors may experience *different trajectories* of memory and executive function symptoms. Identifying higher symptoms subgroups may help to address common underlying characteristics that can be targeted for intervention and guidance.

Cognitive symptoms can result from chemotherapy [12, 13], but have also been associated with surgery [7], other adjuvant therapies [7, 14, 15], and psychological sequelae of cancer (treatment), including depression and fatigue [9]. No previous study has considered how these factors relate to cognitive symptoms trajectories in working cancer survivors. A better understanding of cognitive symptoms trajectories and associated factors may help clinicians to identify patients at risk for more severe cognitive symptoms trajectories and may guide the development of at-work and RTW programs tailored to their individual experiences.

The study objectives were to identify among working cancer survivors: (1) memory and executive function cognitive symptoms trajectories during the first 18 months after RTW and (2) factors associated with these trajectories, including sociodemographic, clinical, psychological, and work-related factors. We postulated that for memory and executive function symptoms, there would be considerable heterogeneity in the trajectories among working cancer survivors.

## Methods

### Study design and sample

The current study is part of the Work-Life after Cancer (WOLICA) study, a longitudinal cohort examining health-related work functioning among 379 working cancer survivors [4]. In WOLICA, cancer survivors completed questionnaires at baseline (within 3 months after returning to work) and after 6-, 12-, and 18-month follow-up.

Occupational physicians (OPs) consecutively recruited cancer survivors aged 18–65 years who had returned to work after cancer diagnosis. Eligible for participation were cancer survivors who were treated with curative intent and returned to paid work for at least 12 h a week. Cancer survivors were required to have a good command of written and spoken Dutch language. Excluded were cancer survivors with recurrent cancer, treated with palliative intent/hospice care, who had no paid employment for at least 1 year prior to the cancer diagnosis.

### Recruitment

Potentially eligible cancer survivors were identified and informed about the study during an OP visit. The involved OPs were working at three national occupational health services in the Netherlands, responsible for about three million (33%) of the approximately nine million workers in the Netherlands. Between March 2013 and July 2015, 516 interested cancer survivors were contacted for participation in the WOLICA study. After inclusion and exclusion criteria were applied (i.e., 39 cancer survivors were not eligible, 13 could not be reached, and one had died), the baseline questionnaire was sent to 463 cancer survivors. A total of 387 survivors (84%) returned the completed questionnaire. The main reason for refusal to participate was “no time to complete the questionnaire.” Three cancer survivors were excluded after completing the baseline questionnaire because their RTW was more than 3 months ago. A detailed description of the study design and recruitment procedures has been provided elsewhere [4].

### Data sources and linkage

Data from WOLICA were linked with the Netherlands Cancer Registry (NCR) to retrieve objective data on clinical factors, i.e., tumor and treatment characteristics. The NCR is an extensive prospective registry of all incident cancer cases in the Netherlands. Three survivors from the WOLICA study could not be linked to the NCR, because they were diagnosed and treated outside the Netherlands and therefore not included in the NCR. For two cancer survivors, the reason is unknown. Those cases were not included in the study. A total of 379 (98.7%) cancer survivors could be linked with the NCR.

### Measures

#### Cognitive symptoms

Cognitive symptoms were measured at four time-points over 18 months, i.e., at baseline and at 6-, 12-, and 18-month follow-up with the Cognitive Symptom Checklist-Work Dutch version (CSC-W DV) [16]. The CSC-W is a self-report measure of a workers’ capacity to use the skills and knowledge necessary to carry out work-related functions. The 19-item CSC-W DV is a reliable and valid measure of cognitive symptoms in cancer survivors [16] and reflects two distinct cognitive domains. (1) The memory symptoms subscale measures the severity of symptoms experienced by working cancer survivors with remembering. (2) The executive function symptoms subscale measures the frequency of symptoms experienced by working cancer survivors when using new information. All items were rated on a Likert scale that ranges from 0 (never) to 4 (always). Both domains demonstrated high internal consistency: memory symptoms (8 items; α = 0.93) and executive function symptoms (11 items; α = 0.94). Total scores range from 0 to 100, with higher scores indicating more cognitive symptoms. The total score and scores on the subscales were obtained by summing the scores on each item, divided by the number of items. The average score is multiplied by 25. When 20% or more of the items were missing, the scale score was set to missing [16]*.*

#### Sociodemographic factors

Sociodemographic factors at baseline comprised sex (male; female), age (in years), and educational level. Educational level was categorized into low (i.e., primary, junior secondary vocational, and junior general secondary education), medium (i.e., senior secondary vocational education and senior general secondary education), and high (i.e., higher professional education, college, and university).

#### Clinical factors

Clinical and treatment-related data were obtained through record linkage with the NCR. Clinical factors included prior cancer diagnosis (yes; no), previous chemotherapy treatment (yes; no), and extent of disease (EoD). EoD was assessed with the EoD-system, based on the US National Cancer Institute Surveillance, Epidemiology, and End Results (SEER) program [17]. Cancer was classified into four categories at the time of diagnosis: (1) localized, i.e., the cancer is limited to the organ of origin, with no sign that the cancer has spread; (2) regional, i.e., the cancer has spread beyond the limits of the organ of origin to nearby lymph nodes, tissues, or organs; (3) distant, i.e., the cancer has spread to distant parts of the of the body; and (4) unknown.

Based on NCR data, the following treatment categories were distinguished: (1) local treatment (i.e., surgery and/or radiotherapy) and (2) systemic therapy (i.e., chemotherapy and/or other systemic therapy using hormonal therapy or targeted therapy, exclusively or in combination with surgery and/or radiotherapy). Finally, undergoing treatment (yes; no) and time between cancer diagnosis and RTW for at least 12 h per week (in months) were assessed in WOLICA.

#### Psychological factors

Psychological factors included fatigue and depressive symptoms. Fatigue was assessed using the 8-item “fatigue severity” subscale of the Checklist Individual Strength [18] (e.g., “I feel tired”). Response options were rated on a seven-point scale (1 = “Yes, that is true” to 7 = “No, that is not true”). Total scores were derived by summing the scores on each item*.* Total scores range from 8 to 56, with higher scores indicating a higher fatigue level. The internal consistency (α = 0.88) is considered good [16]. Depressive symptoms were assessed by the Patient Health Questionnaire-9 (PHQ-9) [19]. The PHQ-9 is a 9-item self-report inventory screening the presence and severity of depression for non-psychiatric settings, corresponding to the Diagnostic and Statistical Manual of Mental Disorders (DSM-IV) criteria of major depression. Response options were rated on a three-point scale (0 = “Not at all” to 3 = “Nearly every day”). Total scores range from 0 to 27, with higher scores indicating higher levels of depressive symptoms. The internal consistency (α = 0.88) is considered good [16].

#### Work-related factors

Work-related factors comprised job type (i.e., manual; non-manual; both manual and non-manual) and psychosocial work environment factors. Psychosocial work environment factors included quantitative job demands (2 items, α = 0.80), work tempo (2 items, α = 0.58), and job control (2 items, α = 0.80) measured with the Copenhagen Psychosocial Questionnaire (COPSOQ) [20]. Response options were rated on a five-point scale (0 = “Never/hardly ever” to 4 = “Always”). Total scores were obtained by summing the items. Total scores ranged from 0 to 8, with higher scores indicating more quantitative job demands, higher work tempo, and high job control.

#### Statistical analyses

First, descriptive data analysis on baseline sample characteristics was conducted. Second, group-based trajectory modeling (GBTM) [21] was used to identify separate trajectories of memory and executive function symptoms over time. GBTM [21] simultaneously estimates multiple trajectories rather than a single population mean, as is the case for traditional regression or growth-curve models. This model also provides the capacity for analyzing the effect of covariates on the probability of group membership. We used the Stata plugin *Traj* [21] for estimating group-based trajectory models, which fits finite (discrete) mixture models to longitudinal data using the maximum-likelihood method.

One to five trajectories solutions were analyzed to determine the optimal number of groups. In each model, the dependent variable was either memory symptoms or executive function symptoms, and the independent variable was time (time 1–4). Memory symptoms and executive function symptoms were modeled as a censored normal distribution. Fit indices, in combination with theoretical interpretability, guided the final model selection. We used the Bayesian information criterion (BIC) and Akaike information criterion (AIC) to test one to five trajectories. Higher BIC and AIC values indicate better model fit [22]. In the second stage of the model search process, we redefined the model by altering the orders of the trajectories. We determined whether each trajectory was best fit by linear terms or intercept only (i.e., constant) to select the most parsimonious model. After model selection, associations of baseline sociodemographic, clinical, psychological, and work-related factors with cognitive symptoms trajectories were investigated by entering all factors together into the GBTM. We converted each parameter to an odds ratio.

## Results

### Sample characteristics

Of the 379 cancer survivors, 63.1% were women, and the mean age at baseline was 50.7 years (*SD* = 8.5; Table 1). The mean time between diagnosis and RTW was 7.4 months (*SD* = 6.5). Nearly half of the cancer survivors (46.4%) had breast cancer, followed by colon cancer (12.7%), hematological cancer (10.0%), and male reproductive cancers (9.2%) (Table 1). More than half of cancer survivors (57.1%) received systemic therapy. Two-thirds (63.9%) of the cancer survivors had completed their treatment at baseline. Cancer survivors reported a mean fatigue score of 30.1 (*SD* = 11.3, min–max = 8–56). Cancer survivors had a mean depression score of 4.7 (*SD* = 3.7, min–max = 0–24). Two-thirds (63.1%) of the cancer survivors reported work accommodations, often working fewer hours/week and working with an adjusted work schedule.

**Table 1:** Participant characteristics (*N* = 379)

	*n*	Mean (*SD*) or %
Age in years (*n* = 375)		50.7 (8.5)
Sex (*n* = 379)		
Male	140	36.9
Female	239	63.1
Education (*n* = 378)		
Low	103	27.2
Medium	128	33.8
High	147	38.8
Marital status (*n* = 378)		
Married/cohabitating	301	79.4
Single/divorced/separated	77	20.3
Type of job (*n* = 377)		
Manual	46	12.1
Non-manual	219	57.8
Both manual and non-manual	112	29.6
Tumor type/diagnosis (*n* = 379)		
Breast cancer	176	46.4
Colon cancer	48	12.7
Hematologic cancer	38	10.0
Male reproductive cancer	35	9.2
Skin cancer	15	4.0
Lung cancer	14	3.7
Gynecological cancer	13	3.4
Head and neck cancer	11	2.9
Gastrointestinal cancer	10	2.6
Urological cancer	10	2.6
Endocrine cancer	4	1.1
Bone, cartilage, and soft tissue cancer	2	0.5
Central nervous system cancer	1	0.3
Eye cancer	1	0.3
Other localizations	1	0.3
Extent of disease at diagnosis (*n* = 261)		
Local	123	32.5
Regional	101	26.7
Distant	11	2.9
Unknown	26	6.9
Treatment (*n* = 370)		
Local treatment	102	26.9
Systemic therapy	268	70.7
Treatment completed (*n* = 378)		
Yes	242	63.9
No	136	35.9
Prior cancer diagnosis (*n* = 379)		
No	352	92.9
Yes	27	7.1
Prior chemotherapy (*n* = 379)		
No	365	96.3
Yes	14	3.7
Time diagnosis to RTW in months (*n* = 357)		7.4 (6.5)
Depressive symptoms at baseline^1^ (*n* = 377)		4.7 (3.7)
Fatigue at baseline^1^ (*n* = 377)		30.1 (11.3)
Psychosocial work environment factors at baseline^1^		
Quantitative job demands	379	1.3 (14.6)
Tempo	379	3.8 (10.5)
Job control	379	3.3 (12.4)
Cognitive symptoms		
Memory symptoms at baseline^1^	374	32.2 (19.2)
Memory symptoms 6 months after RTW	328	31.7 (19.6)
Memory symptoms 12 months after RTW	302	32.5 (19.7)
Memory symptoms 18 months after RTW	277	32.0 (19.8)
Executive function symptoms at baseline^1^	352	19.6 (15.7)
Executive function symptoms 6 months after RTW	314	19.2 (15.7)
Executive function symptoms 12 months after RTW	290	19.3 (16.2)
Executive function symptoms 18 months after RTW	270	19.7 (16.9)

Note: ^1^Within the first 3 months after return to work; *RTW*, return to work

### Cognitive symptoms trajectories

**Fig. 1:**
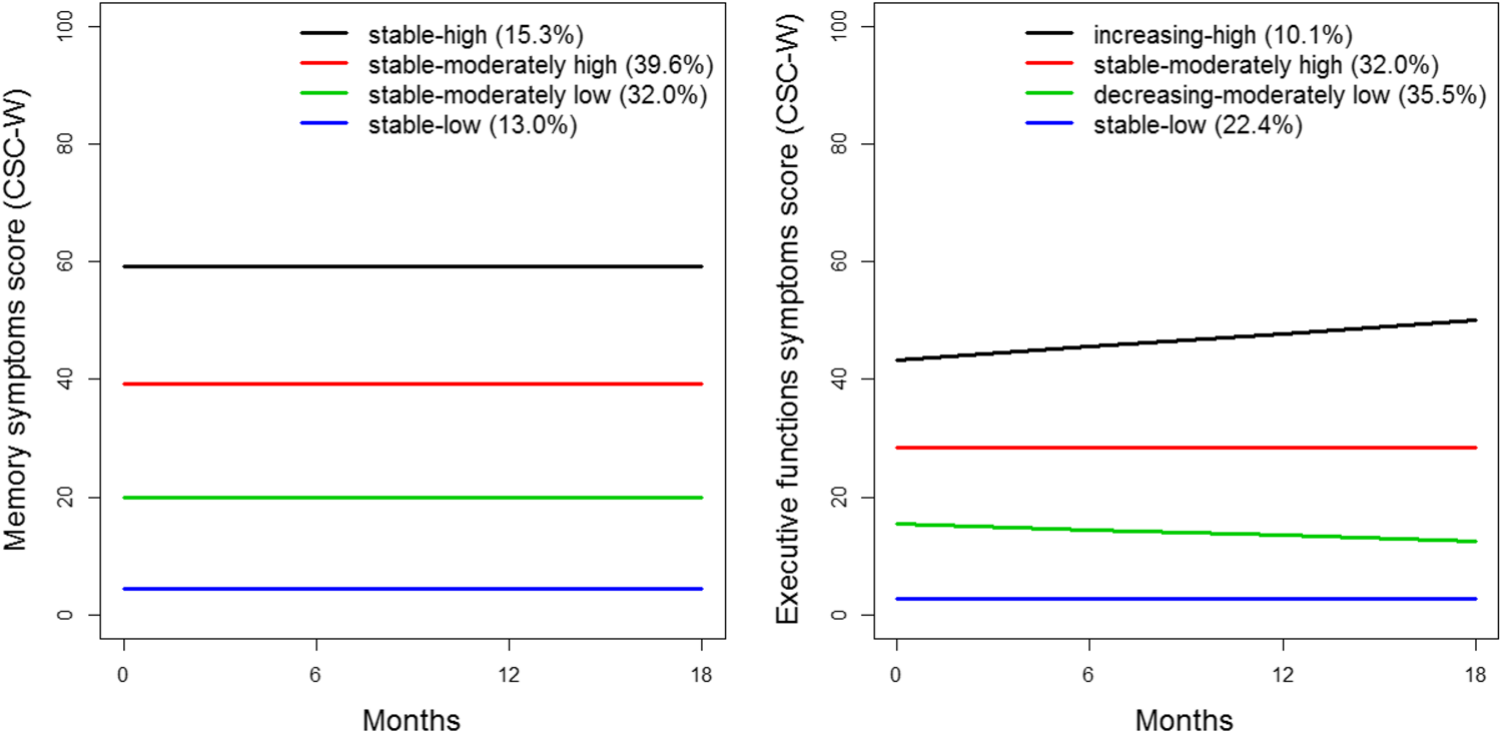
Four-class model for memory symptoms and executive function symptoms

For memory symptoms, the five-class model had the highest BIC and AIC (Supplementary Table 1). When comparing the four- and five-class models, the extra class of the five-class model showed the same stable pattern compared to the other classes. Based on the conceptual meaningfulness, the four-class model was preferred over the five-class model. For executive function symptoms, the five-class model had the highest AIC, but contained a small group (1.3%, *n* = 5) (Supplementary Table 2). Based on this, the four-class model was preferred over the five-class model. The four latent trajectory classes that emerged are presented in Fig. 1. All memory symptoms trajectories remained stable over 18-month follow-up at different severity levels: “stable-high” (15.3% of the sample), “stable-moderately high” (39.6%), “stable-moderately low” (32.0%), and “stable-low” (13.0%).

Likewise, four executive function symptoms trajectories at different severity levels were identified. A small group (10.1%) of working cancer survivors represents with “increasing-high” executive function symptoms over time. The majority of cancer survivors display “stable-moderately high” (32.0%) and “decreasing-moderately low” (35.5%) executive function symptoms over time. The “stable-low” (22.4%) trajectory displays low and stable executive function symptoms over time.

### Factors associated with cognitive symptoms trajectories

For memory symptoms, cancer survivors with a longer time from diagnosis to RTW were more likely to be part of the “stable-high” (OR = 1.11; 95% CI = 1.02–1.21), “stable-moderately high” (OR = 1.09; 95% CI = 1.01–1.17), and “stable-moderately low” (OR = 1.09; 95% CI = 1.01–1.17) memory symptoms trajectories compared to the “stable-low” trajectory (= reference category) (Table 2). Similarly, cancer survivors reporting higher levels of depressive symptoms were more likely to be part of the “stable-high” (OR = 1.74; 95% CI = 1.40–2.15), “stable-moderately high” (OR = 1.49; 95% CI = 1.24–1.80), and “stable-moderately low” (OR = 1.24; 95% CI = 1.01–1.52) trajectories. Cancer survivors reporting more quantitative work demands were more likely to be part of the “stable-high” (OR = 1.32; 95% CI = 1.00–1.74) trajectory compared to the “stable-low” memory symptoms trajectory.

**Table 2:** Parameter estimates for risk factors associated with each memory symptoms trajectory group. The stable-low trajectory was chosen as the reference category

	Stable-moderately low			Stable-moderately high			Stable-high		
	OR	95% CI	*p* value	OR	95% CI	*p* value	OR	95% CI	*p* value
*Sociodemographic factors*									
Age	1.04	(0.99–1.09)	0.0887	1.03	(0.99–1.08)	0.1778	1.01	(0.95–1.08)	0.7426
Gender									
Male	Ref			Ref			Ref		
Female	1.31	(0.60–2.85)	0.4923	2.14	(0.96–4.76)	0.0617	3.04	(0.91–10.15)	0.0702
Education									
High	Ref			Ref			Ref		
Medium	1.12	(0.46–2.68)	0.8067	0.54	(0.21–1.35)	0.1878	2.51	(0.76–8.34)	0.1330
Low	0.67	(0.26–1.75)	0.4143	0.52	(0.21–1.27)	0.1495	0.57	(0.13–2.46)	0.4554
*Clinical factors*									
Type of cancer treatment									
Local treatment	Ref			Ref			Ref		
Systemic therapy	1.07	(0.43–2.67)	0.8885	0.96	(0.38–2.42)	0.9253	2.21	(0.61–7.98)	0.2279
Time between diagnosis and RTW^a^	**1.09**	**(1.01**–**1.17)**	**0.0348**	**1.09**	**(1.01**–**1.17)**	**0.0282**	**1.11**	**(1.02**–**1.21)**	**0.0158**
Treatment completed									
Yes	Ref			Ref			Ref		
No	0.93	(0.39–2.20)	0.8669	0.96	(0.41–2.24)	0.9205	0.72	(0.22–2.35)	0.5864
*Psychological factors*									
Depressive symptoms	**1.24**	**(1.01–1.52)**	**0.0382**	**1.49**	**(1.24–1.80)**	** < 0.0001**	**1.74**	**(1.40–2.15)**	** < 0.0001**
Fatigue	0.99	(0.95–1.03)	0.5289	0.99	(0.95–1.03)	0.7352	1.02	(0.95–1.09)	0.5799
*Psychosocial work environment factors*									
Quantitative job demands	1.15	(0.94–1.41)	0.1690	1.02	(0.99–1.06)	0.2258	**1.32**	**(1.00–1.74)**	**0.0470**
Tempo	1.05	(0.85–1.29)	0.6550	0.93	(0.83–1.04)	0.1915	0.96	(0.84–1.09)	0.4927
Job control	0.90	(0.73–1.11)	0.6575	0.89	(0.73–1.10)	0.2864	0.95	(0.71–1.27)	0.7244

Note: Odds ratios and 95% confidence intervals were presented. *RTW*, return to work; ^a^In months

For executive function symptoms, older cancer survivors were more likely to be part of the “increasing-high” trajectory (OR = 1.09; 95% CI = 1.01–1.18) compared to the “stable-low” trajectory (= reference category) (Table 3). Cancer survivors with a longer time from diagnosis to RTW were more likely to be part of the “increasing-high” (OR = 1.14; 95% CI = 1.04–1.24) and “stable-moderately high” (OR = 1.09; 95% CI = 1.00–1.18) executive function symptoms trajectories. Cancer survivors reporting higher levels of depressive symptoms were more likely to be part of the “increasing-high” (OR = 1.80; 95% CI = 1.41–2.28), “stable-moderately high” (OR = 1.58; 95% CI = 1.27–1.96), and “decreasing-moderately low” (OR = 1.33; 95% CI = 1.07–1.66) trajectories. Cancer survivors reporting more quantitative work demands were more likely to be part of the “stable-moderately high” (OR = 1.22; 95% CI = 1.00–1.48) executive function symptoms trajectory.

**Table 3:** Parameter estimates for risk factors associated with each executive function symptoms trajectory group. The stable-low trajectory was chosen as the reference category

	Decreasing-moderately low			Stable-moderately high			Increasing-high		
	OR	95% CI	*p* value	OR	95% CI	*p* value	OR	95% CI	*p* value
*Sociodemographic factors*									
Age	1.03	(0.99–1.08)	0.1466	1.04	(1.00–1.10)	0.072	**1.09**	**(1.01–1.18)**	**0.0283**
Gender									
Male	Ref			Ref			Ref		
Female	1.73	(0.76–3.92)	0.1906	0.95	(0.41–2.18)	0.9044	2.28	(0.58–9.00)	0.2397
Education									
High	Ref			Ref			Ref		
Medium	0.75	(0.30–1.88)	0.5438	1.38	(0.55–3.45)	0.4904	1.07	(0.29–3.96)	0.9165
Low	0.69	(0.27–1.79)	0.4464	0.87	(0.31–2.41)	0.7889	1.09	(0.28–4.18)	0.9000
*Clinical factors*									
Type of cancer treatment									
Local treatment	Ref			Ref			Ref		
Systemic therapy	0.86	(0.34–2.18)	0.7528	1.01	(0.38–2.65)	0.9882	1.03	(0.26–4.15)	0.9652
Time between diagnosis and RTW^a^	1.07	(0.99–1.16)	0.0748	**1.09**	**(1.00–1.18)**	**0.0388**	**1.14**	**(1.04–1.24)**	**0.0038**
Treatment completed									
Yes	Ref			Ref			Ref		
No	0.78	(0.33–1.85)	0.5713	0.97	(0.40–2.37)	0.9473	1.81	(0.54–6.00)	0.3345
*Psychological factors*									
Depressive symptoms	**1.33**	**(1.07–1.66)**	**0.0114**	**1.58**	**(1.27–1.96)**	** < 0.0001**	**1.80**	**(1.41–2.28)**	** < 0.0001**
Fatigue	0.99	(0.94–1.03)	0.5062	0.97	(0.93–1.01)	0.1794	0.99	(0.93–1.05)	0.6520
*Psychosocial work environment factors*									
Quantitative job demands	1.05	(0.99–1.11)	0.1363	**1.22**	**(1.00–1.48)**	**0.0456**	1.00	(0.96–1.05)	0.8361
Tempo	0.90	(0.78–1.03)	0.1169	0.86	(0.74–1.01)	0.0677	0.98	(0.86–1.13)	0.8133
Job control	0.96	(0.76–1.20)	0.7092	0.87	(0.70–1.09)	0.2256	0.88	(0.69–1.11)	0.2878

Note: Odds ratios and 95% confidence intervals were presented. *RTW*, return to work; ^a^In months

## Discussion

We identified four cognitive symptoms subgroups for memory and executive function among working cancer survivors. Trajectories had distinct and graded levels of cognitive symptoms at baseline and remained relatively stable over the 18-month follow-up. Higher cognitive symptoms trajectories were associated with higher levels of depressive symptoms, a longer time between diagnosis and RTW in months, more quantitative work demands, and older age. To the best of our knowledge, this is the first study to report distinct cognitive symptoms trajectories in working cancer survivors. This research has important clinical implications, as discussed below.

Earlier studies showed that average cognitive symptoms scores remain stable and persistent over time [9, 11]. The findings of a recent study in cancer survivors who are non-durable work-disabled suggest that cognitive symptoms persist over time despite some decrease between 2 and 4 years after the first day of sick leave [23]. Similarly, in this study, we identified that the general pattern of cognitive symptoms trajectories in working cancer survivors was relatively stable over the first 18 months after RTW. However, we found a large heterogeneity of cognitive symptoms severity within working cancer survivors. That is, in our sample, 58.9% of the survivors reported high memory symptoms at baseline (= return to work). These symptoms did not diminish in the 18-month follow-up period. Likewise, 42.1% reported high executive functioning symptoms at baseline that remained high over time. Finding this heterogeneity can be explained by the use of GBMT, by which we have been able to identify distinctive clusters of individual trajectories within working cancer survivors.

To the best of our knowledge, earlier studies did not examine factors associated with cognitive symptoms trajectories in working cancer survivors. Paquet et al. [3] showed that depressive symptoms could be linked to self-report measures of cognitive functioning in cancer survivors. Similarly, we found higher levels of depressive symptoms in cancer survivors in “stable-high” and “increasing-high” cognitive symptoms trajectories compared to “stable-low” trajectories. A longer time from diagnosis to RTW was associated with higher cognitive symptoms trajectories. This is consistent with Dorland et al. [4], finding that cancer survivors with persistently high work functioning trajectories reported less time between diagnosis and RTW compared to cancer patients in moderate to high and persistently low trajectories. Hence, later RTW might be indicative of poorer functioning. Further, older age and more quantitative work demands were associated with higher cognitive symptoms trajectories. Knowledge of the factors associated with cognitive symptoms trajectories may be useful in identifying cancer patients who returned to work at risk for enduring cognitive symptoms. This information may support priority setting and cancer patient performance targets to be used by health care professionals or employers in sustainable employment.

### Strengths and limitations

A strength is the longitudinal design with repeated measurements of cognitive symptoms at baseline, 6, 12, and 18 months after RTW. Data from all four measurement points were available for the majority (80.5%) of cancer survivors. Cohort data were linked to detailed objective clinical data from the NCR. A validated measure of cognitive symptoms in working cancer survivors was employed [16]. The results are generalizable to working cancer survivors. The sample is highly educated and consists of mostly breast cancer patients. The sample might be biased toward patients who returned to work after cancer diagnosis and treatment with better cognitive functioning. In contrast, patients with poorer outcomes might be underrepresented. Caution should be taken in generalizing to the broader population of cancer survivors. Our results need to be corroborated in more heterogeneous studies.

### Implications for practice and research

Our findings suggest that the presence of enduring cognitive symptoms should play a more prominent role in the clinical and occupational health care systems during the RTW process. Clinicians and OPs should assess cognitive symptoms at baseline after RTW to identify cancer survivors with higher symptomatic and lower functioning trajectories, either with self-report measures, such as the CSC-W [16], or with neuropsychological tests [24]. Employers should ensure that accommodations are available for employees with cognitive symptoms. Cancer survivors need accurate information on the potential occurrence of cognitive symptoms and assistance with symptoms management. Potential interventions include individual guidance, psycho-education, cognitive strategy training, and fatigue management. More and more initiatives are implementing these general cognitive rehabilitation programs (e.g., the Internet-based cognitive rehabilitation for WORking Cancer survivors [i-WORC]) for oncology patients [25]. The factors associated with cognitive symptoms trajectories in working cancer survivors may help clinicians identify survivors at risk for more severe cognitive symptoms to develop personalized treatment. General population norm data for the CSC-W are needed to support interpretation of the clinical significance of cognitive symptoms. Finally, future studies on cognitive symptoms in working cancer survivors should incorporate pre-treatment cognitive assessment.

### Conclusion

In this study, we have provided suggestive evidence for different stable memory and executive function symptoms trajectories in the 18 months after RTW. The identification of trajectories of memory and executive function symptoms may allow clinicians to monitor or predict cognitive symptoms severity levels. More baseline depressive symptoms were found in cancer survivors in “stable-high” and “increasing-high” cognitive symptoms trajectories compared to “stable-low” trajectories. This knowledge may help to lay out priorities and target efforts of healthcare professionals and the workplace to aid cancer survivors after RTW.

## Supplementary Information

Below is the link to the electronic supplementary material.

Supplementary file1 (DOCX 19 KB)
