# Supplementary material for: Trajectories of cognitive symptoms and associated factors in cancer survivors after return to work: an 18-month longitudinal cohort study
Source: J Cancer Surviv. 2022 Mar 21;17(2):290–9. doi: 10.1007/s11764-022-01190-3 (PMC10036271; doi:10.1007/s11764-022-01190-3)
Supplement: Supplementary file 1 — Supplementary file1 (DOCX 19 KB) [file 11764_2022_1190_MOESM1_ESM.docx]

**Trajectories of cognitive symptoms and associated factors in cancer survivors after return to work: An 18-month longitudinal cohort study**

**Journal of Cancer Survivorship**

Authors: Johanna K. Ehrenstein^1,2^, Sander K.R. van Zon^1^, Saskia F.A. Duijts^,3,4^, Roy E. Stewart^1^, Josué Almansa^1^, Benjamin C. Amick III^5^, Sanne B. Schagen^2,6^, Ute Bültmann^1^

**Name, City, and Affiliation of all authors:**

^1^ University of Groningen, University Medical Center Groningen, Department of Health Sciences, Community and Occupational Medicine, Hanzeplein 1, PO Box 30.001, 9700 RB, Groningen, The Netherlands

^2^ The Netherlands Cancer Institute, Division of Psychosocial Research and Epidemiology, Plesmanlaan 121, 1066 CX, Amsterdam, The Netherlands

^3^ Vrije Universiteit Amsterdam, Amsterdam UMC, Amsterdam Public Health Research Institute, Department of Public and Occupational Health, Van der Boechorststraat 7, 1081 BT, Amsterdam, The Netherlands

^4^ Netherlands Comprehensive Cancer Organisation (IKNL), Department of Research and Development, Godebaldkwartier 419, 3511 DT, Utrecht, The Netherlands

^5^ University of Arkansas for Medical Sciences, Fay W. Boozman College of Public Health, Department of Epidemiology, Little Rock, Arkansas, United States of America

^6^ University of Amsterdam, Department of Psychology, Nieuwe Achtergracht 129-B, 1018 WT, Amsterdam, The Netherlands

**Corresponding author:**

Johanna K. Ehrenstein, MSc, MSc

University of Groningen, University Medical Center Groningen

Department of Health Sciences, Community and Occupational Medicine

Hanzeplein 1, PO Box 30.001, 9700 RB

9713 AV Groningen

The Netherlands

Telephone: +31 645514967

E-mail: j.k.ehrenstein@umcg.nl

**Supplementary Tables**

*Supplementary Table 1.*

*Goodness-of-fit indicator values for each model class for memory symptoms*

| Model | K | Order | BIC (N=1281) | BIC (N=377) | AIC |
| --- | --- | --- | --- | --- | --- |
| 1 | 1 | 1 | -5416.04 | -5414.20 | -5408.30 |
| 2 | 2 | 1,1 | -5144.07 | -5140.40 | -5128.61 |
| 3 | 3 | 1,1,1 | -5039.04 | -5033.54 | -5015.84 |
| 4 | 4 | 1,1,1,1 | -5005.39 | -4998.05 | -4974.46 |
| **5** | **4** | **0,0,0,0** | **-4991.85** | **-4986.96** | **-4971.23** |
| 6 | 5 | 1,1,1,1,1 | -4991.30 | -4982.13 | -4952.63 |

Note: K, the number of groups. The order indicates whether the trajectory was fit with

a constant (0) or linear function. BIC, Bayesian information criterion;

AIC, akaike information criterion. Higher BIC and AIC values indicate better models.

The preferred 4-class model is presented in bold.

*Supplementary Table 2.*

*Goodness-of-fit indicator values for each*

*model class for executive function symptoms*

| Model | K | Order | BIC (N=1226) | BIC (N=374) | AIC |
| --- | --- | --- | --- | --- | --- |
| 1 | 1 | 1 | -4680.23 | -4678.45 | -4672.56 |
| 2 | 2 | 1,1 | -4470.98 | -4467.42 | -4455.65 |
| 3 | 3 | 1,1,1 | -4395.01 | -4389.67 | -4372.01 |
| 4 | 4 | 1,1,1,1 | -4371.63 | -4364.51 | -4340.96 |
| **5** | **4** | **0,1,0,1** | **-4364.87** | **-4358.93** | **-4339.31** |
| 6 | 5 | 1,1,1,1,1 | -4371.36 | -4362.46 | -4333.03 |

Note: K, the number of groups. The order indicates whether the trajectory was fit with

a constant (0) or linear (1) function. BIC, Bayesian information criterion;

AIC, akaike information criterion. Higher BIC and AIC values indicate better models.

The preferred 4-class model is presented in bold.
